# Medical Society Proceedings

**Published:** 1870-09

**Authors:** M. W. Wilcox, Wm. E. Henry

**Affiliations:** Pres.; Mattoon, Ill.; Sec.; Mattoon, Ill.


MATTOON, ILL., May 25, 1870.

Esculapian Society of the Wabash Valley met pursuant to adjournment; Dr. M. W. Wilcox, President.

The following members were present: Drs. Albin, Deming, Chambers, Johnson, Miller, Massie, St. Clair, Ringland, Swafford, Mosley, Steel, Pearman, Todd, Washburn, Willien, and Henry.

The minutes of the last meeting at Paris, Ill., were read and approved.

Dr. Willien's report on surgery was first in order, which was a paper occupying nearly an hour in its reading, and consisted of a statement of cases, and their treatment, together with many practical and original ideas. He made mention of the singular phenomenon, not usually observed, concerning the cessation of growth in the finger or toe-nails in cases of fracture of bones of the arms or legs; which fact it would be well to remember.

A case of irreducible strangulated brural hernia of right side. Kelotomy, and recovery.

Mrs. P——, resident of Effingham, Ill., aged 39 years, mother of five children, hernia of ten years standing, was suddenly taken sick, in July, 1869, with all the symptoms of strangulated hernia. All ordinary remedies failing, Drs. St. Clair, Lecrone, and he (the operator) decided to operate, as the only means of saving life. The symptoms were fast becoming more aggravated, vomiting of stercoraceous matter, etc., etc. 7th inst. Patient chloroformed. Tumor, size of large hen's egg, much inflamed, and very painful to the touch.

The operation was conducted after the usual manner, very little blood was lost; hernia reduced; wound closed with silk thread, ice applied in after dressing, opium internally.

Suppuration of wound on third day, a simple dressing then of carbolized glycerine and simple cerate. The bowels were moved freely on the seventh day by a dose of castor oil. Patient made an immediate recovery, and a radical cure was the result.

Next case was that of peri-uterine abscess. Supposed cause, perimetritis. Operation, and recovery.

Mrs. H. aged 27 years, mother of two children, in act of lifting a tub was seized with severe pain in back, followed by excessive uterine hemorrhage, with temporary loss of consciousness. She was four months gone in pregnancy, and miscarriage followed. The pain in back and sacro-lumbar region continued, with frequent stranguary and tenesmus of bowels. The preceding physician in the case (an irregular) had pronounced the cause of all the trouble a uterine polypus. When the patient desired its removal, he put her under the influence of chloroform and made traction on the neck of the womb; but, wearied out, he declared it would have to be removed by "escoratics." This course of treatment, fortunately, was never attempted.

Dr. Willien saw her first in Feb. 1869, after an illness of three years. Symptoms, face pale with expressions of anxiety, pulse weak and frequent, chilling, at night cold and abundant perspiration, pain in abdomen and lower bowels, constipation.

On examination, a tumor, size of large infant's head, extending above the pubis about three inches; was painful, fluctuating, and slightly movable.

Exam. per vaginal. Cervix inclined to right, its posterior labia enlarged; os partly dilated and granulated, from which a sero-sanguinous fluid issued of a very offensive character. The uterus was lower in the vagina than normal; distinct fluctuation in cul de sac.

Per rectum exam. Tumor easily defined and fluctuation evident. Ques. Was it an intra-uterine disease involving its normal structure, a malignant growth, or a peri-uterine abscess? The introduction of the sound showed a declination to the left. The neck was dilated by tent sponge, and uterus found exempt from tumor or disease; he then concluded it to be an accumulation of fluid between the peritoneal covering and tissues proper; was it serous, blood or pus? The history of the case led him to think it pus.

March 30. Patient anesthetized, a small trocæ was plunged through the vaginal cul de sac and a rush of white inodorous pus, confirmed his diagnosis. An injection of carbolic acid and rain water was then used, narcotic poultices applied over the abdomen, and opium internally to relieve pain. Sulph. quinia and other tonics were freely administered. On fourth day, tumor began to enlarge again, although the discharge continued per vaginam. On examination per rectum, the pressure ruptured the membranes and a discharge of pus followed the finger. She now began to improve with a good appetite, and after a few months is again about her ordinary domestic work.

Third case was that of a fracture of the lower jaw in two places, by kick of horse; one spicula of bone protruding through into the mouth. The interesting part about this case was the application of a new splint (invented by himself) of vulcanized rubber, made to fit the jaw inside and externally. After six weeks the splints were removed with entire satisfaction.

Dr. Willien, at the close of his report, presented the Society with two new splints for the forearm and the leg; these, for their simplicity and cheapness, as well as their real merits, were generally approved. Any physician, living near a tinner, could have them readily made. A trough-like arrangement, with an expansion for the hand and a perforated septum for the arm or leg to rest on, through which all the superabundant water and effete materials could pass, thence off at one end. A complete dressing could be effected without any disturbance of the limb.

After the reading of this paper, it was discussed by Drs. Swafford, Massie, Chambers, and others.

Dr. Swafford thought the excessive use of cold water, by a large part of the profession, and as recommended by some medical authors, not a very commendable treatment; many times producing bad results.

Dr. Massie's report on midwifery was next in order. This document consisted of the doctor's own views and how he would conduct labor. It gave evidence of much experience and thought, was practical and original, written for the benefit of the young men in the profession, and abounded in ideas not to be found in books. The part referring to ergot, elicited debate from some few members, advocates pro and con, as heretofore. The report was well received and was requested for publication.

The semi-annual address was delivered by Dr. Swafford of New Goshen, Ind., and was listened to attentively. It covered much of the history of the profession from 450 B. C. down to the merest charlatan or Indian doctor of the present, showing, with reason, why impostors did, and the regular profession did not, advertise.

May 26. Society met according to adjournment at 8 o'clock, A. M.

Dr. Chambers, of Charleston, Ill., reported a case. Mr. C——, farmer, age 40 years, June 1, 1869, was driving into his barn on a load of door-frames, was caught between the load and roof. Was carried from his barn to his house, a short distance. It was found, on examination, that the last dorsal vertebra was dislocated, one of the ribs on left side fractured, and lower extremities paralyzed. Patient was chloroformed, and extension made and the luxation reduced; one or two of the processes of the vertebra above were thought to be fractured; a compress, with bands around the waist, secured the vertebra in place. Morphine was given to relieve the pain; in a few days, bromide potass. was commenced and continued in large doses. Urine had to be drawn by catheter. In six weeks strychnia was given, but, not agreeing with the patient, nux vomica was substituted. This remedy seemed to fail, and an electric battery was used for some months.

May 21, 1870. Saw Mr. C—— to day, was sitting up in a rocking-chair, in which position he had been for some four hours. He weighed, when he received his injury, 160 pounds, was reduced in November last to 100 pounds, now weighs 125 pounds; he presents a healthy appearance, appetite sufficient, tongue clean, skin soft and warm to his toes; can now make partial flexion and extension of the left leg and slight motion with the right. Sensibility to the knee almost perfect; legs are proportionately smaller than his body; is nearly always admonished when it is necessary to urinate, yet cannot restrain it from passing; bowels rather torpid, thinks he can tell when he is going to have a passage, yet not always; has had, during the past winter, partial erections of the penis; pulse in sitting posture, 80 degrees, recumbent, 78; the case at this time is about "statu quo." It is interestin g from the fact of its being the third on record. The doctor promised to keep watch over the case, and report to the Society in the fall as to its progress or condition. He then reported a case of gastrodynia as cured by sub. nit. bismuth.

A card was then read before the Society issued by one of its members: "Confidential Instructions given to the Married." "Dr. Davis has the only reliable and harmless preventive, (thereby interdicting the crime of abortion.) He also treats successfully all forms of private diseases, gonorrhœa, syphilis, leucorrhœa, painful menstruation, stricture, impotence, seminal weakness, etc." Call at the ——, room No. —, or address, box 451, Des Moines, Iowa, with 2 three cent stamps for reply"

The following was written on card : " 25$\frac{00}{100}$."

He was expelled by a unanimous vote of the Society.

The following gentlemen were assigned as essayists at the fall meeting:

Dr. A. J. Miller, Paris, Ill., on Baths Dr. J. B. Hedges, Clinton, Ind., Obscure Mararial Diseases. Dr. J. F. Price, Charleston, Ill., Improvements in Therapeutic Agents. Dr B. F. Swafford, New Goshen, Ind., Wounds of the Knee Joint. Dr. H. H. Deming, Mattoon, Ill., Substitutes for Quinia. Dr. H. F. Harper, Marion, Ind., Best Manner of Building with a view to Ventilation. Dr. J. H. Apperson, Fillmore, Ill., the Sources of the Gastric Juice. Dr. J. R. Hinkle, Sullivan, Ind., Spinal Irritation.

The president then appointed the following gentlemen chairmen of standing committees:

Dr. G W. Albin, Neoga, Ill, Surgery. Dr. L L. Todd, Paris, Ill., Practical Medicine Dr. Charles S. Johnson, New Goshen, Ind., Epidemics. Dr. E. B. Cannon, Tuscola, Ill., Midwifery. Dr. R. L. Walstons, Paris, Ill., Indigenous Botany.

Committee of Arrangements for next meeting: Drs. Apperson, Cannon, and Brinton.

Tuscola, Ill., was decided upon as the place for the fall meeting.

Dr. J M. Hinkle, of Mattoon, Ill., brought up the question of paralysis, in connection with the building of the piers in the Mississippi river at St. Louis. The men having to work in a compressed atmosphere (under a pressure of about three atmospheres) were after a time (some in a few hours, others after a day or a week,) paralyzed. This led him to conclude that there was yet a frequent cause for this disease, not fully recognized by the profession. He thought if the external pressure exerted by the atmosphere was a cause of paralysis, as it seemed clearly to be in the above cases, then probably it acted conjointly with other common or recognized causes in everyday life, and his remedy would be to send such cases to more elevated localities, mountainous countries.

The time was so far gone that the debate on this question was cut short, and the Society adjourned to meet on the fourth Wednesday in October, 1870.

M. W. WILCOX, *Pres.*

WM E. HENRY, *Sec.*

